# Detection of fusion transcripts in the serum samples of patients with hepatocellular carcinoma

**Published:** 2019-05-21

**Authors:** Yan-Ping Yu, Allan Tsung, Silvia Liu, Michael Nalesnick, David Geller, George Michalopoulos, Jian-Hua Luo

**Affiliations:** ^1^Department of Pathology and Surgery, University of Pittsburgh School of Medicine, Pittsburgh, PA 15261, USA; ^2^Current address: Department of Surgery, Ohio State University School of Medicine, Columbus, Ohio 43210, USA

**Keywords:** serum detection, fusion transcript, cell-free RNA, HCC

## Abstract

Hepatocellular carcinoma is one of the most lethal cancers in the United States. Early detection of the disease is crucial for reducing the mortality of this malignancy. Recently, we identified a panel of fusion genes present in several types of human cancers, including hepatocellular carcinoma. Among 8 fusion genes, MAN2A1-FER, TRMT11-GRIK2 and CCNH-C5orf30 appear most frequently in hepatocellular carcinoma samples. In this study, we showed that the fusion transcripts of MAN2A1-FER, CCNH-C5orf30 and SLC45A2-AMACR were detected in the serum samples of liver cancer patients as circulating cell-free RNA. The distributions of these gene fusion RNA fragments largely matched those of the primary HCC samples. In contrast, the sera of all healthy individuals free of human malignancies were shown to be negative for these fusion genes. These results suggest that gene fusion RNA is frequently shed from liver cancer cells. The detection of serum cell-free fusion transcripts may provide a new approach to aid in the diagnosis, follow-up or therapy of liver cancers.

## INTRODUCTION

Human cancer is one of the most frequent causes of death in the United States. In 2018, the mortality rate of cancer reached 606,880 in the US [1], making cancer the second most lethal cause of death after cardiovascular diseases [2]. Hepatocellular carcinoma (HCC) is one of the most lethal malignancies, accounting for more than 31,000 deaths in the US alone [1]. The five-year survival rate for HCC is approximately 18%. Only pancreatic adenocarcinoma and glioblastoma multiforme have lower survival rates [3]. The development of early detection methods and effective treatment for HCC is urgently needed to reduce the mortality of this disease.

Treating liver cancer in the early clinical stages offers a significant advantage for therapeutic options and a better prognosis [4]. Currently, surgical resection, ablation and liver transplant are the most effective approaches to treating early-stage HCC [5, 6]. HCC patients treated with these approaches typically survive long-term and can even be considered cured of the disease. However, patients with late clinical stage HCC without similar options of surgical intervention usually survive less than a year. Thus, early detection of HCC is crucial for reducing the mortality of liver cancer. Recently, we identified a panel of 8 fusion genes in human cancers [7–9]. Some of these fusion genes were shown to be present in a large proportion of HCC cancer samples [7, 10]. The mechanisms underlying these gene fusions are chromosomal translocation and rearrangement [8–10]. The presence of these fusion transcripts in liver cancer samples indicates that translocation and chromosomal rearrangement are common in liver cancer cells. To investigate the utility of these fusion transcripts in detecting liver cancer, we performed TaqMan qRT-PCR on the RNA extracted from cell-free serum. The results suggest that many of these fusion genes are detectable as cell-free circulating RNA.

## RESULTS

One of the hallmarks of genomes of human cancer is chromosomal rearrangement and translocation [11, 12]. Previously, we identified a panel of fusion genes in prostate cancer samples from patients who experienced poor clinical outcomes. Subsequent analyses showed that many of these fusion genes are present in a variety of human cancers, including liver cancer [7, 10]. To investigate whether these fusion transcripts are detectable in the sera of HCC patients, we analyzed the presence or absence of 8 fusion genes in 118 serum samples from HCC patients and individuals free of malignant tumors. As shown in Table 1, all serum samples from individuals free of malignancies were negative (0/14) for the fusion transcripts of all the fusion genes. In contrast, 83.7% (87/104) of the serum samples from HCC patients were positive for at least one fusion transcript. Interestingly, all serum samples obtained from HCC patients with non-alcoholic steatohepatitis etiology (n=20) were positive for at least one fusion gene (p=0.019): 100% (20/20) versus 77.6% (59/76). The fusion gene is also more likely present in the serum samples from HCC patients with steatohepatitis background (p=0.02): 100% (20/20) versus 78.5% (62/79). Interestingly, multiple fusion transcripts (at least 2) detected in the serum are associated with moderate differentiation of HCC (42.4% [14/33] versus 9.5% [2/21] for all other category, p=0.014).

**Table 1:** Fusion transcripts detection in the sera of HCC patients

Clinical characteristics	Fusion gene positive	Fusion gene negative
**HCC patients**		
**Age:**		
40s	5	0
50s	22	2
60s	33	11
70s	20	1
80s	4	3
**Etiology:**		
HCV	44	8
HBV	6	2
Ethanol	34	9
NASH	20	0
Other	4	4
**Background liver:**		
Cirrhosis/fibrosis	81	17
Steatosis	12	4
Steatohepatitis	20	0
**Recurrent status:**		
Recurrent	20	2
Non-recurrent	50	11
**Response to therapy:**		
Responsive	31	8
Progressive	42	6
**Pathology grade:**		
Poorly differentiated	5	0
Moderately differentiated	28	5
Well differentiated	18	6
**Death**	40	7
**Alive**	42	9
**Healthy individuals**	0	14

Upon analyzing individual fusion transcripts, we determined that MAN2A1-FER was frequently detected in the sera of HCC patients, reaching 78.8% (82/104, Table 2). SLC45A2-AMAMCR occurred at a frequency of 31.7% (33/104), while CCNH-C5orf30 occurred at a frequency of 10.6% (11/104). All HCC patients with NASH were positive for MAN2A1-FER in their serum samples (20/20), indicating a strong association between MAN2A1-FER and the etiology of NASH (p=0.005).

**Table 2:** Frequency of individual fusion transcript detected in the sera of HCC patients

Clinical features	*MAN2A1-FER*	*TRMT11-GRIK2*	*MTOR-TP53BP1*	*CCNH-C5orf30*	*KDM4B-AC011523.2*	*SLC45A2-AMACR*	*TMEM135-CCDC67*	*LRRC59-FLJ60017*
All HCC patients	83/104	0/104	0/104	11/104	0/104	33/104	0/104	0/104
**Ages:**								
>80s	11/15	0/15	0/15	0/15	0/15	6/15	0/15	0/15
70s	21/22	0/22	0/22	2/22	0/22	7/22	0/22	0/22
60s	36/48	0/48	0/48	8/48	0/48	11/48	0/48	0/48
50s	12/14	0/14	0/14	1/14	0/14	5/14	0/14	0/14
40s	2/2	0/2	0/2	0/2	0/2	2/2	0/2	0/2
**Etiology:**								
HCV	42/54	0/54	0/54	8/34	0/54	19/54	0/54	0/54
HBV	7/8	0/8	0/8	1/8	0/8	1/8	0/8	0/8
Ethanol	31/43	0/43	0/43	2/43	0/43	11/43	0/43	0/43
NASH	21/21	0/21	0/21	4/21	0/21	7/21	0/21	0/21
Other	2/5	0/5	0/5	0/5	0/5	0/5	0/5	0/5
**Background liver:**								
Cirrhosis/fibrosis	81/101	0/101	0/101	12/101	0/101	28/101	0/101	0/101
Steatosis	11/16	0/16	0/16	0/16	0/16	7/16	0/16	0/16
Steatohepatitis	20/21	0/21	0/21	3/21	0/21	7/21	0/21	0/21
**Recurrent status:**								
Recurrent	20/22	0/22	0/22	2/22	0/22	5/22	0/22	0/22
Non-recurrent	50/63	0/63	0/63	10/63	0/63	20/63	0/63	0/63
**Response to therapy:**								
Responsive	29/38	0/38	0/38	5/38	0/38	12/38	0/38	0/38
Progressive	42/49	0/49	0/49	7/49	0/49	16/49	0/49	0/49
**Pathology differentiation grade:**								
Poor	4/5	0/5	0/5	0/5	0/5	3/5	0/5	0/5
Moderate	26/34	0/34	0/34	5/34	0/34	5/34	0/34	0/34
Well	18/24	0/24	0/24	2/24	0/24	3/24	0/24	0/24
**Death**	39/48	0/48	0/48	5/48	0/48	17/48	0/48	0/48
**Alive**	41/51	0/51	0/51	5/51	0/51	15/51	0/51	0/51
**Healthy person**	0/14	0/14	0/14	0/14	0/14	0/14	0/14	0/14

**Figure 1:**
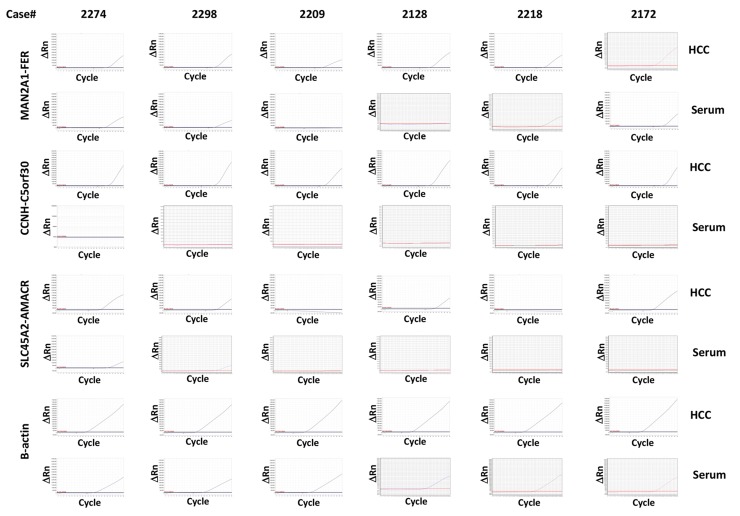
Detection of MAN2A1-FER, CCNH-C5orf30 and SLC45A2-AMACR in HCC samples and the corresponding serum samples Six cases of HCC and matched serum samples were analyzed for the presence of transcripts of MAN2A1-FER, CCNH-C5orf30 and SLC45A2-AMACR using TaqMan qRT-PCR. The results of β-actin were used as normalization controls. Assays were performed twice independently. Sanger sequencing was performed on 20% of all positive samples.

To determine whether the HCC samples from the same patients were positive for these fusion genes, six liver cancer samples from these HCC patients were analyzed. As shown in Table 3 and Figure 1, MAN2A1-FER and CCNH-C5orf30 were positive in all six HCC samples, while 4 of 6 HCC samples were positive for SLC45A2-AMACR. All positive serum samples corresponded to the matched positive HCC samples, indicating that the source of the seral fusion transcripts was the liver cancer. Two serum samples were negative for MAN2A1-FER, while the matched HCC samples were positive for the fusion gene. Similarly, two HCC samples positive for SLC45A2-AMACR had matched serum samples that were negative for the same fusion gene. Even though TRMT11-GRIK2 and CCNH-C5orf30 were present in all six HCC samples, the transcripts of these fusion genes were undetectable in the sera of the same patients. Half of the HCC samples were also positive for LRRC59-FLJ60017, but the fusion transcript was not detected in the matched serum samples. Based on these results, MAN2A1-FER appears to be the most sensitive marker for serum detection of HCC (4/6 or 67%). SLC45A2-AMACR ranks second (2/4 or 50%), while TRMT11-GRIK2 and CCNH-C5orf30 are the most insensitive markers (0/6 or 0%). These results suggest that these fusion transcripts have different levels of detectability in the blood, probably due to differences in the sensitivity of these RNA sequences to circulating RNAses.

**Table 3:** Fusion gene detection in serum versus matched HCC tissue

Case No.	*MAN2A1-FER*	*TRMT11-GRIK2*	*MTOR-TP53BP1*	*CCNH-C5orf30*	*KDM4B-AC011523.2*	*SLC45A2-AMACR*	*TMEM135-CCDC67*	*LRRC59-FLJ60017*
**2274**								
HCC tissue	+	+	-	+	-	+	-	-
Serum	+	-	-	-	-	+	-	-
**2298**								
HCC tissue	+	+	-	+	-	+	-	-
Serum	+	-	-	-	-	+	-	-
**2209**								
HCC tissue	+	+	-	+	-	-	-	+
Serum	-	-	-	-	-	-	-	-
**2128**								
HCC tissue	+	+	+	+	-	+	-	+
Serum	-	-	-	-	-	-	-	-
**2218**								
HCC tissue	+	+	-	+	-	-	-	-
Serum	+	-	-	-	-	-	-	-
**2172**								
HCC tissue	+	+	-	+	+	+	-	+
Serum	+	-	-	-	-	-	-	-

## DISCUSSION

Cancer-specific gene fusions are the result of chromosomal rearrangement and translocation [8]. Many gene fusion events are not specific to one type of cancer. Indeed, most of the fusion genes we discovered in prostate cancer were later detected in a variety of human malignancies, including HCC [7, 10]. MAN2A1-FER and SLC45A2-AMACR belong to a class of fusion genes with gain of function. The MAN2A1-FER gene fusion generates a new chimera protein in which the C-terminal glycoside hydrolase of mannosidase alpha, class 2A, member 1 (MAN2A1) is replaced with an intact tyrosine kinase domain from FER [9, 10]. The new chimera protein exhibits an almost 4-fold increase in tyrosine kinase activity compared with that of the native FER, and the chimera protein is translocated to the Golgi apparatus [10]. The resulting chimera protein transforms immortalized cells into cancer through the ectopic phosphorylation of growth factor receptor [10]. MAN2A1-FER was detected in 15% of human HCC samples, and this fusion protein was shown to be a driver of mouse liver cancer [10]. SLC45A2-AMACR was also detected in a lung cancer cell line [13] and urothelial carcinoma [14], in addition to prostate cancer [9, 15]. To our knowledge, this is the first report showing that SLC45A2-AMACR is present in HCC and the corresponding serum samples. AMACR is a racemase responsible for branch fatty acid metabolism, while SLC45A2 is a solute transporter. The fusion generates a chimera protein such that 5 transmembrane domains of SLC45A2 are removed from its C-terminus and replaced with an intact racemase domain from AMACR. The overexpression of AMACR was shown to be associated with the aggressive behavior of multiple human cancers [16–25]. *In vitro*, AMACR was shown to increase cell growth and proliferation [26].

CCNH-C5orf30 and TRMT11-GRIK2 belong to a class of fusion genes with loss of function. CCNH-C5orf30 was detected in 37% of HCC samples [10], while TRMT11-GRIK2 was detected in 12.9% of HCC samples [7]. The gene fusion of CCNH-C5orf30 produces a truncation of the H5′ and HC domains of cyclin H (CCNH), which is an important cell cycle regulator for the progression to mitosis [27, 28], and an independent C5orf30 protein. The truncated CCNH in the fusion gene is defective in binding with cdk7 [29] and may be defective in its transcriptional activity and promotion of the cell cycle. A more dramatic loss of function is identified in the TRMT11-GRIK2 gene fusion; the TRMT11-GRIK2 gene fusion eliminates the open-reading frame of GRIK2, which is a potential tumor suppressor [30, 31], and produces a large truncation of TRMT11, which is a tRNA methyltransferase [32, 33]. Thus, the fusion event is equivalent to the structural deletion of both TRMT11 and GRIK2 genes. The deletion of TRMT11 reduces the stability of tRNA and may therefore adversely impact the protein translation of cancer cells, while the loss of GRIK2 may promote the growth of cancer cells. All these gene fusion events may play important roles in the development of human liver cancer.

The abnormal chromosomal recombination that generates these fusion genes is cancer-specific and is absent in normal tissues [8]. The frequent presence of these fusion transcripts in the serum samples of HCC patients suggests that these RNAs are derived from liver cancer cells. Three lines of evidence support the hypothesis that the fusion RNA fragments detected in the serum are shed from liver cancer cells. First, the pattern of fusion transcript distribution in the serum completely overlaps that of the corresponding liver cancer samples, i.e., there is no fusion transcript that is positive in the serum but negative in the matched liver cancer sample. Second, all serum samples from healthy individuals are negative for these fusion transcripts. Normal individuals do not produce these fusion genes. Third, the normalized quantities of the detected fusion transcripts in the serum are generally 4- to 16-fold lower than those detected in the corresponding HCC samples, suggesting the shedding of RNAs from a fraction of cancer cells.

Early detection of HCC is probably the most effective way to reduce the mortality of the disease, due to the availability of many effective surgical treatments. Unfortunately, at the time of diagnosis, the HCCs of many patients are at the advanced stages, eliminating many options for curing the disease [4]. Currently, the primary means of diagnosing HCC relies on radiology imaging of liver cancers. The screening of HCC for patients with cirrhosis and chronic liver diseases involves biannual ultrasonography [4]. This screening method may be combined with a seral test for α-fetoprotein or other imaging analyses, such as MRI, CT and contrast-enhanced ultrasound, if a suspicious nodule appears. The presence of fusion transcripts from HCC cancer cells in the serum may represent a new approach for detecting liver cancer. Since the test is minimally invasive, it can be employed regularly in conjunction with ultrasound screening for patients with chronic liver disease or cirrhosis. When a suspicious nodule is detected, the presence of a fusion transcript may help to confirm the diagnosis. All 14 healthy individuals negative for the fusion transcript had no known liver disease and were cancer-free. The high frequency of fusion genes in liver cancer implies that gene fusion in the cancer genome is an early event for liver cancer development. It is of interest to investigate whether these fusion genes are also present in some of the HCC precursor lesions such as NASH. So far, the fusion transcripts appear cancer-specific. The serum detection of these fusion genes may provide a sensitive follow-up test for patients undergoing surgical resection or liver transplant to monitor the recurrence of liver cancer. A new therapeutic approach was recently developed to target the chromosomal breakpoints of these fusion genes using the CRISPR-cas9 gene editing system [34]. This approach led to the partial remission of xenografted human liver cancers when the animals were treated with reagents targeting the breakpoint of the MAN2A1-FER fusion gene [34]. As a result, the detection of these fusion genes in HCC may have significant therapeutic implications. The utilization of cell-free fusion RNA in the circulation as tumor markers may provide an important means for early detection, follow-up and therapeutic guidance for the management of HCC patients.

## MATERIALS AND METHODS

### Tissue samples

The 124 tissue specimens and serum samples used in this study consisted of 6 hepatocellular carcinoma samples from HCC patients, 104 serum samples from HCC patients and 14 serum samples from non-cancerous patients. These samples were obtained from the University of Pittsburgh Tissue Bank in compliance with institutional regulatory guidelines. The informed consent exemptions and protocol were approved by the Institution Review Board of the University of Pittsburgh. All serum samples and hepatocellular carcinoma samples were fresh-frozen and stored at -80°C. Some cases have multiple etiologies, pathological features, and backgrounds. They were classified multiple times. When statistical analyses were performed, however, the duplication was excluded from the analyses. HCC cases that do not contain the specific clinical information were also excluded from the analyses.

### RNA extraction, cDNA synthesis and detection of fusion genes

The procedures for RNA extraction, cDNA synthesis and the detection of fusion genes were described previously [35–49]. Briefly, total RNA was extracted using Trizol to lyse the cells in the cancer tissues (Invitrogen, CA, USA). First strand cDNA was synthesized using ~2 µg of RNA from each sample, random hexamers and Superscript II™ (Invitrogen, Inc, CA, USA) at 42°C for 2 hours. One microliter of each cDNA sample was used for the TaqMan PCR reactions with 50 heat cycles, as follows: 94°C for 30 seconds, 61°C for 30 seconds, and 72°C for 30 seconds. The following primers and probes were used: MAN2A1-FER (AGCGCAGTTTGGGATACAGCA/CTTTAATGTGCCCTTATATACTTCACC; TaqMan probe, 5’/56-FAM/TCAGAAAC A/ZEN/GCCTATGAGG GAAATT/3IABkFQ/3’), SLC45A2-AMACR (TTGAT GTCTGCTCCCATCAGG/CAGCTGGAGTTTCTCCAT GAC; TaqMan probe, 5'-/56-FAM/AAGAGGGCA/ZEN/TGGTAGTGGAGGC/3IABkFQ/-3'), CCNH-C5orf30 (AAAGTTATTTATCAGAGAGTCTGATGCTG/CTGTT CTACTCCAGGTATTTTCATTATATC; TaqMan probe, 5'-/56-FAM/ACAGGCAAG/ZEN/TTCTGTTCTCTTTC AGCA/3IABkFQ/-3'), mTOR-TP53BP1 (TGATAGA CCAGTCCCGGGATG / CCACTGACATTCCCAGA ACAAG; TaqMan probe, 5'-/56-FAM/ TGTCAGCCT/ZEN/GTCAGAATCCAAGTCAAG/3IABkFQ/-3'), TRM T11-GRIK2 (GCGCTGTCGTGTACCCTTAAC / GAAT GCAAGTTCCTCAGCTCC; TaqMan probe, 5'-/56-FAM/ CGGAACTCC/ZEN/AGATGCTCCTGCG/3IABkFQ/ -3'), LRRC59-FLJ60017 (GTGACTGCTTGGATG AGAAGC / CCCTCCTCTGGTTTGTTGTTG; TaqMan probe, 5'-/56-FAM/CAGTGTGCA/ZEN/AACAAGGT GACTGGAAG/3IABkFQ/-3'), TMEM135-CCDC67 (CAGCTGTCATGGAAGTTCAGAC / CCTCATTCT TTCCTGCTCAGAG; TaqMan probe, 5'-/56-FAM/AGTTCCTTT/ZEN/TAAGACTCACCAAGGGCAA/3IA BkFQ/-3'), KDM4- AC011523.2 (AGACC ACCTTCGCCTGGCAC / TCTCTCTCAGATCCAG GCTTG; TaqMan probe, 5'-/56-FAM/ACAGCATCA/ZEN/ACTACCTGCACTTTGGG/3IABkFQ/-3'), and β-actin (ACCCCACTTCTCTCTAAGGAG / GCAATGCTATC ACCTCCCCTG; TaqMan probe, 5'-/56-FAM/CCA GTCCTC/ZEN/TCCCAAGTCCACAC/3IABkFQ/-3’). The PCR reactions were performed in a thermocycler (Eppendorf Realplex™ thermocycler). A negative control and synthetic positive control were included in each batch of reactions. The PCR products were gel-purified and Sanger-sequenced for 20% of the positive samples.

## Abbreviations

AC011523.2: Homo sapiens chromosome 19 clone LLNLF-214C7
AMACR: Alpha-methylacyl-CoA racemase
C5orf30: Chromosome 5 open-reading frame 30
CCNH: Cyclin H
FER: Fez related tyrosine kinase
FLJ60017: Homo sapiens cDNA FLJ60017
GRIK2: Glutamate ionotropic receptor kainate type subunit 2
HCC: Hepatocellular carcinoma
KDM4B: Lysine demethylase 4B
LRRC59: Leucine rich repeat containing 59
MAN2A1: Mannosidase class 2 member A1
mTOR: Mechanistic target of rapamycin kinase
qRT-PCR: quantitative reversed transcription polymerase chain reaction
SLC45A2: Solute carrier family 45 member 2
TP53BP1: Tumor protein p53 binding protein 1
TRMT11: tRNA methyltransferase 11 homolog
